# Different Management Options for Transplant Ureteral Obstructions within an Inguinal Hernia

**DOI:** 10.1155/2016/4730494

**Published:** 2016-04-10

**Authors:** Felix Cheung, Merrit Marion Debartolo, Leonard Michael Copertino, April Adams Szafran, Chelsea Caron Estrada, Patrick Gerard Lynch, Frank Sam Darras

**Affiliations:** ^1^Department of Urology, Stony Brook University Medical Center, Stony Brook, NY 11794, USA; ^2^Department of Surgery, Stony Brook University Medical Center, Stony Brook, NY 11794, USA; ^3^Department of Transplant, Stony Brook University Medical Center, Stony Brook, NY 11794, USA; ^4^Division of Nephrology, Stony Brook University Medical Center, Stony Brook, NY 11794, USA

## Abstract

Ureteral obstruction secondary to an inguinal hernia with transplant ureteral component is an extremely rare entity with only several case reports found in literature. In all previously reported cases, management of the obstruction involved temporary drainage with ureteral stenting or nephrostomy tube placements followed by delayed definitive repair. We present two case reports, here one being the first one managed by immediate definitive repair via ureteral reimplant and herniorrhaphy and a second case by delayed definitive repair after percutaneous nephrostomy tube placement. Both patients continued to do well postoperatively with normalization of renal function on follow-up.

## 1. Introduction

Transplant ureteral kidney obstruction is not an uncommon occurrence. Different causes of urinary obstruction in kidney transplants may include ischemia to distal ureteral segment, injury, stones, crossing vessels, lymphocele, or ureteral kinking [1]. Ureteral herniation through an inguinal hernia is an extremely rare cause of urinary obstruction, with only several reported cases in literature. Here, we present 2 cases of transplant ureteral herniation and the different approach to management. Both patients continued to do well with normalization of kidney function to baseline after relief of obstruction.

## 2. Case Report

### 2.1. Case  1

**Figure 1:**
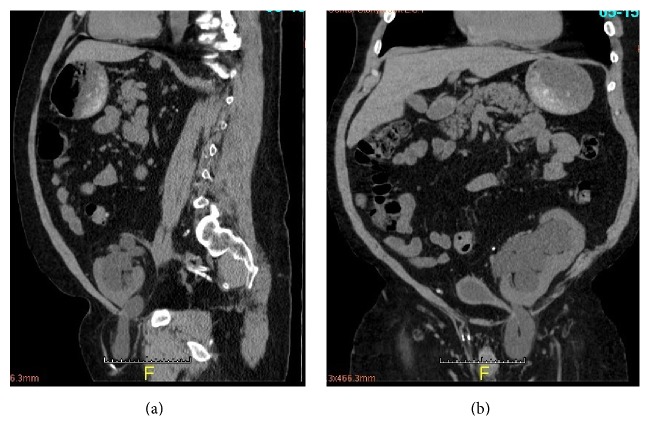
Noncontrast CT abdomen pelvis in sagittal (a) and coronal (b) views revealing transplant ureter within inguinal hernia resulting in hydroureteronephrosis.

A 44-year-old obese white male with a history of ESRD secondary to childhood hemolytic-uremic syndrome, with post-DDRT status in 1998, presented with AKI with serum Cr of 3.4 mg/dL from his baseline of 2 mg/dL. He denied any symptoms. Exam was significant for a palpable left inguinal hernia. US revealed moderate to severe hydronephrosis of the transplanted kidney with nonvisualization of the distal ureter, and no bladder abnormalities. Despite IV hydration and adequate bladder emptying, the patient's urine output was marginal and Cr remained elevated at 3.1 mg/dL. A noncontrast CT of the abdomen and pelvis was obtained (Figure 1). A left inguinal hernia was seen to contain the distal transplant ureter with resultant proximal moderate to severe hydronephrosis of the transplant kidney, evident of obstruction.

**Figure 2:**
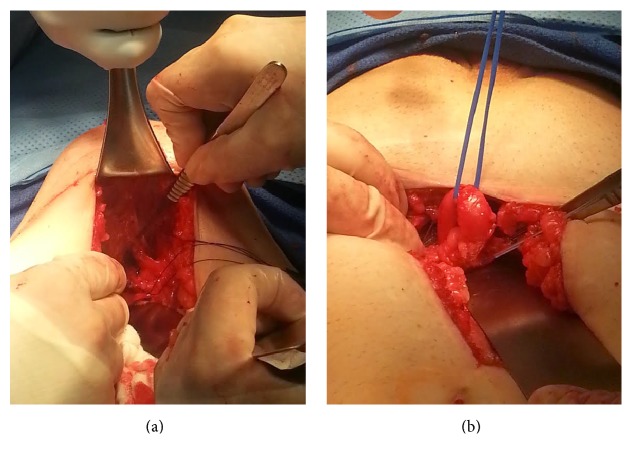
Hernia defect seen within the preperitoneal space (a); vessel loop around the redundant transplant ureter reduced from the hernia (b).

The patient was taken to the operating room the next day for surgical exploration. A 2 × 3 cm defect between the internal oblique fascia and the shelving edge of the inguinal ligament was exposed, and a highly redundant transplant ureter was reduced (Figure 2). The defect was repaired via preperitoneal approach with nonabsorbent sutures in interrupted fashion. The redundant transplant ureter was transected to an appropriate length and reimplanted into the anterior bladder wall over a ureteral stent. The patient tolerated the procedure well without immediate complications. He was discharged on post-op day 2. At two-week follow-up, his Cr had improved to 1.71 mg/dL and remained stable 8 months post-op.

### 2.2. Case  2

An 88-year-old male with PCKD and ESRD had LRT in 2001. During his hospitalization for pneumonia, he developed AKI with his Cr elevated from 1.5 mg/dL to 2.6 mg/dL. A renal transplant US revealed moderate to severe hydronephrosis with hydroureter; however, the distal portion was not visualized. A CT cystogram was performed which demonstrated vesicoureteral reflux into the collecting system and incarceration of dilated ureter within a right inguinal hernia. The patient was deemed not an ideal surgical candidate in the setting of acute pneumonia. He underwent emergent placement of nephrostomy tube to the transplanted kidney with subsequent improvement of patient's Cr to 1.28 mg/dL. Two months later, he underwent definitive repair of the hernia. A low midline incision was used for exploratory laparotomy and, within the preperitoneal space, the hernia defect was encountered with transplant ureter herniation. The ureter was reduced and herniorrhaphy was performed in preperitoneal fashion. The redundant transplant ureter was revised and reimplanted into the bladder with ureteral stent placement. The existing nephrostomy tube was removed. Postoperatively, the patient's Cr improved to 1.03 mg/dL. He was discharged from hospital on post-op day 7 after normalization of bowel function. At his 4-month follow-up, he continues to do well with normal kidney function (Cr 1.06 mg/dL).

## 3. Discussion

Common causes of transplant ureteral obstructions include ureteral strictures, malignancy, and calculous disease [1]. Renal transplant hydroureteronephrosis has many etiologies, including ureteral obstruction, reflux, infection, and rejection. Hydronephrosis is usually identified by US while nuclear renal imaging with Lasix can further asses for obstruction. Cystoscopy with ureteral stenting or percutaneous nephrostomy placement can provide the usual first line options to relieve obstruction [1, 2]. In rare instances, inclusion into an inguinal hernia can also cause transplant ureteral obstruction with only three other case reports in literature. Nonetheless, inguinal hernias should be considered in the differential diagnosis in the setting of transplant ureteral obstruction as they represent a common disease process in general and urologic surgical patients and one that urologists and transplant surgeons are able to treat.

Factors that predispose to transplant ureteral obstruction include obesity and redundant ureteral length. Anterior positioning of the transplant ureter in relation to the spermatic cord also is a risk factor [1], although this was not the case for either of our two cases. Regardless of the predisposing influences, ureteroinguinal hernias can be categorized in three different ways depending on their proximity to peritoneum: paraperitoneal (next to peritoneal hernia sac), peritoneal (through peritoneal hernia sac), and extraperitoneal (not associated with a hernia sac altogether) [3]. Concomitant repair of inguinal hernias via a preperitoneal approach during pelvic urologic surgery, particularly with radical retropubic prostatectomy and radical cystoprostatectomy, has been described in the urologic literature [2].

All three prior reports of transplant ureteral obstruction due to inclusion into an inguinal hernia were managed by temporary placement of percutaneous nephrostomy tubes or ureteral stenting prior to delayed definitive surgery as with the scenario presented here in case 2 [1, 4, 5]. However, we also demonstrated that immediate and definitive repair of the ureteral obstruction with herniorrhaphy and ureteral reimplant is a feasible option. This case represents the first report of successful immediate repair of transplant ureteral obstruction. Given our patient's favorable outcome as measured by clinical status, rapid recovery, and Cr improvement, we show that when promptly diagnosed with the appropriate index of suspicion, immediate definitive herniorrhaphy with ureteral reconstruction is a feasible option for selected patients with transplant ureteral obstruction secondary to inclusion into an inguinal hernia.

## Abbreviations

AKI: Acute kidney injury
Cr: Creatinine
CT: Computed tomography
DDRT: Deceased donor renal transplantation
ESRD: End stage renal disease
IV: Intravenous
LRT: Living donor related renal transplantation
PCKD: Polycystic kidney disease
US: Ultrasound.
